# Delicious but Immoral? Ethical Information Influences Consumer Expectations and Experience of Food

**DOI:** 10.3389/fpsyg.2019.00843

**Published:** 2019-04-24

**Authors:** Beth Armstrong, Aaron Meskin, Pam Blundell-Birtill

**Affiliations:** ^1^School of Philosophy, Religion and History of Science, University of Leeds, Leeds, United Kingdom; ^2^School of Psychology, University of Leeds, Leeds, United Kingdom

**Keywords:** ethics, sustainability, food choice, taste, flavor, Fairtrade, aesthetics

## Abstract

It has been suggested that information about ethically relevant factors in production can affect both the expectation and experience of foods. However, evidence on these issues is inconsistent. We begin by discussing recent philosophical work on the interaction of ethical and aesthetic values in the domain of food, which is inspired by a similar debate about art. Some philosophers have suggested that ethical factors in production that leave a ‘trace’ on a product, i.e., make a perceivable difference to it, will affect the aesthetic quality of the food. There has also been the suggestion that these sorts of ethical/aesthetic interactions may vary across different kinds of food. In two studies we examined the expected experience and the actual experience of eating various foods, when participants had been given ethically relevant information about those foods. We examined people’s ethical values and the effect that had on the ratings. We found strong evidence to suggest that ethically relevant information affects expected experience of food and that the valence of the information is a significant factor. We found an effect of ethical values on expectations of food. Most notably, we found evidence that suggests that ‘trace’ may be a relevant factor mediating the effect of ethically relevant information on expectations and experience of food. Future research should further explore the factor of trace, look at the effect of ethical information in a wider range of foods, and investigate these phenomena in distinct populations.

## Introduction

Food, like art and many other things that human beings care about, can be valued (found good or bad) in a variety of ways—ethically, nutritionally, financially, aesthetically, and so on (Budd, 1996). The plurality of values that we care about enriches our lives, but it also presents us with challenging situations where we face conflicts in our values (Connors et al., 2001). A course of action seems good from a financial perspective but bad from an ethical perspective. How should we act? Ethically, we hope!

Many of us face these sorts of conflicts as consumers and eaters on a regular basis (Furst et al., 1996). “It’s probably harmful to the environment but tastes great.” “It’s bad for the planet but yummy.” “It’s delicious but unethical.” It is striking how similar such sentiments are to the way people talk about art, literature and music. “It’s a great film but it’s racist.” “It’s a beautiful painting but probably sexist.” “The novel is brilliant but immoral.” And so on.

Such statements seem to imply that the conflicting values are *independent* of one another. On the one hand, the food and film are great. They are valuable from gustatory and cinematic perspectives respectively. But on the other hand, the food and film are both bad from the ethical perspective. And these different perspectives seem to have nothing to do with one another. The film is aesthetically good *but* morally flawed. The food is delicious *but* ethically bad. This is an ‘autonomist’ approach (Clavel-Vazquez, 2018). Autonomism may seem like a tempting view, especially in the domain of food. Ethical values and deliciousness may seem to have nothing to do with one another.

But perhaps the values in question are not independent of one another. For example, it might be that a moral flaw can make a film worse. Philosophers of art and literature have been fascinated by this possibility, and there has been a great deal of research in the area in recent years arguing for a range of subtle positions on the matter (Kieran, 2006). ‘Moralists’ hold that some works of art (e.g., Leni Riefenstahl’s *Triumph of the Will*, which served in part as Nazi propaganda) are aesthetically flawed precisely because of their immorality (Gaut, 1998). However, some philosophers are ‘immoralists’ or ‘contextualists’ who hold that there are contexts in which moral flaws can *enhance* a work’s aesthetic value (Eaton, 2012).

Inspired by the analogy between the food and art cases, a number of philosophers have attempted to adapt theories designed to make sense of the interaction of morality and aesthetics in the artistic domain to the domain of food (Korsmeyer, 2012; Liao and Meskin, 2018). These philosophers have argued that (1) food has both aesthetic and moral dimensions (i.e., it can be valued both aesthetically and ethically), and (2) that these dimensions interact in various ways.

Korsmeyer (2012) argues for a moralist view about food. She suggests that some moral flaws in food production or preparation, ones which leave a ‘trace’ on food items such as the cruel treatment of animals, will negatively affect the aesthetic value of those items. Conversely, other factors, which do not leave such a trace (such as the use of nets for catching tuna which also kill dolphins), will not have such an effect.

Liao and Meskin (2018) agree that the ethical status of food may impact gustatory experience and that trace may be relevant to this effect. They argue that the aesthetic value of food, which they understand largely in terms of deliciousness, may be affected by its moral status. But Liao and Meskin argue for a contextualist position. When the moral flaw found in the food is necessary for achieving a highly valued feature, such as in the production of ikizukuri sashimi, where the fish are cut while alive in order to maximize freshness, they argue that this may actually count as improving the aesthetic quality and deliciousness of the food.

Although experimental philosophy is an emerging area of research (Knobe and Nichols, 2017), philosophers do not typically run experiments to test their theories. In two studies, we begin to explore those theories experimentally. Does moral information affect aesthetic judgments of food and, if so, how (i.e., does information about moral flaws in food always have a negative effect on aesthetic judgment or can it sometimes have a positive effect)? Does the existence of what Korsmeyer calls a ‘trace’ matter to this effect? And does context (e.g., the type of food and ethical attitudes) make a difference?

There is evidence that flavor and quality are important in determining food choice (Furst et al., 1996). However, the majority of consumers are also very concerned about the production and processing of foods (Torjusen et al., 2001). Flavor of food is affected by the physical characteristics of the food (Spence and Piqueras-Fiszman, 2014), the genetic characteristics of the consumer (Drayna, 2005), but also by top–down cognitive processes. For example, linguistic labels change the pleasantness of flavors (de Araujo et al., 2005).

There is evidence to support the notion that ethical information about food can influence consumer experience (e.g., Schuldt and Schwarz, 2010; Bratanova et al., 2015). Labeling a food in an ethically relevant way can have a positive effect on consumers’ expectations and perceptions. For example, Sörqvist et al. (2015) reported that banana samples which were labeled as eco-friendly were perceived to have a superior flavor compared to ‘standard’ bananas. Labeling chocolate as Fairtrade increased expected and actual taste intensity and pleasantness, compared to chocolate that was labeled as conventional (Enax et al., 2015). This ‘halo effect’ can lead consumers to expect a product to taste better, be more nutritious and even benefit cognitive function (Magnusson et al., 2001; Sörqvist et al., 2015).

Research tends to compare the effect of positively valenced information about food, such as being told that a product is organic or sustainable (e.g., de Andrade Silva et al., 2017), on the experience (or expectation) of that food, with the experience (or expectation) of standard products, which are either not labeled, or labeled as conventional or regular (e.g., Annett et al., 2008; Linder et al., 2010). Only a handful of studies have explored how negative ethical information impacts consumer expectations and perceptions (Bratanova et al., 2015; Anderson and Barrett, 2016). However, the limited evidence indicates that labeling foods as having negative moral status can negatively impact expectations regarding that food. For example, Anderson and Barrett (2016) reported that beef jerky was liked less when it was labeled as factory farmed, even when that factory farming was given a positive framing.

In line with the philosophical arguments offered by Liao and Meskin, ethical information does not always have the effect that it might be expected to have. Sörqvist et al. (2015) found that there were no effects of labeling water as eco-friendly on judgments of taste. This suggests that it is not the case that the experience of all foods and beverages are affected by ethical information in the same way. In fact, some studies have demonstrated findings in line with the predictions of Liao and Meskin: the experience of some foods, such as fruit, benefits from positive information about their ethical status, whereas the experience of other foods can be negatively influenced by positive ethical information (Bourn and Prescott, 2002; Kihlberg et al., 2005; Bratanova et al., 2015). For example, Lee et al. (2013) found that cookies that were labeled as organic were judged as tasting worse than regular cookies, whereas yogurt labeled as organic was judged as tasting better than regular yogurt, suggesting that ethical, or ethically relevant, effects might depend on food type.

Sometimes the direction of the ethical effect depends upon what is being measured. Schuldt and Hannahan (2013) found that organic foods were rated healthier than standard (non-organic) foods, but they were also rated as less tasty. Additionally, the individual values of the participants impacts the ethical effect. Participants with a high level of environmental concern expected organic foods to be tastier than those who were low in environmental concern, while the consumer’s level of environmental concern did not influence the perceived healthfulness of organic products (Schuldt and Hannahan, 2013).

Following Korsmeyer (2012) and Liao and Meskin (2018), we suspect that one factor which may make a difference to the effect of ethical, or ethically relevant, information on the experience of food is whether the ethical manipulation might be expected to leave a ‘trace’ on the food, that is, have a perceptible impact on it. Ethical attitudes are closely linked to attitudes toward, and consumption of, organic food (Honkanen et al., 2006). If all else was equal, food which is produced organically might be expected to be perceptibly different to food which is not organic. However, food that has been produced by well-paid workers might not be expected to be perceptibly different from food that has been produced by workers living in poverty. If leaving a trace really is a factor, then describing a product as organic might be expected to change the taste experience of the food since food that is organic is thought to be detectably different from food that is non-organic (Magkos et al., 2006). However, describing a product as Fairtrade might not be expected to change the experience of the food since treatment of workers is not believed to make a detectable difference on the quality of the food produced (Lotz et al., 2013).

The present study aims to explore how valence of the information, and ‘trace’ of the ethical manipulation impact participants’ expectations of a food. Extending the existing literature, we aim to understand if, and how, ethical information can influence consumer expectations of products.

## Study 1

The first study examined how consumer product expectations are influenced by ethical information of differing valence and content. As previous research has indicated that some foods may be more susceptible to ethical manipulation than others (Lee et al., 2013), we investigated how ethical information affected the expectations of different foods. (We note that for the remainder of the paper we will follow US Food and Drug Administration practice and use the term ‘food’ to refer to all foods and beverages). Based on the literature discussed above, we formed four hypotheses.

First, we proposed that ethical information will have an effect on the expectations of a product. Second, we predicted that ethical information that leaves a trace will have a bigger effect on expectations than ethical information that does not leave a trace. Third, we anticipated that the valence of the ethical manipulation (e.g., positive, negative) will impact the direction of the change in expectations. Finally, we suggested that those who score highly on measures of ethical attitudes will show a greater impact of ethical manipulations of food expectations. Additionally, as Piazza et al. (2015) demonstrated gender and dietary preferences influence ethical concerns regarding meat consumption, we suspected that different demographic groups (e.g., gender, dietary preference and age) might be differentially affected by ethically relevant product information, but made no specific predictions as to demographic effects.

### Methods

The experiment was pre-registered with the OSF: https://osf.io/nmsx3/.

#### Participants

Two-hundred and ninety-three adults (*M* = 33.6 years, *SD* = 9.3 years, 32% female) who lived in the United Kingdom were recruited using Amazon’s Mechanical Turk. A minimum sample size *N* = 96 was determined for power of 80%, with *f*^2^ = 0.15 (a medium effect size) and alpha = 0.05, using an *a priori* sample size calculator (Soper, 2019). All subjects were paid^1^ for their participation. As cultural differences could impact attitudes toward ethically produced foods (Sörqvist et al., 2016) and taste preferences (Poelman et al., 2008) it was necessary to limit the recruitment to a specific region. The United Kingdom is one of the world’s leading Fairtrade markets (Fairtrade Foundation, 2018), part of the largest organic market (Yussefi and Willer, 2003), and crustacean welfare issues have recently been reported in national media. Hence, United Kingdom consumers are likely to be aware of, and motivated by, the ethical issues surrounding food production.

In order to ensure data quality, the questionnaire included an attention check (Meade and Craig, 2012; Kung et al., 2018). The attention check consisted of a single question which instructed participants to select a radio button located at the bottom of the page, and to ignore a 7-point Likert scale to the right of the question. If a response on the Likert scale was selected, or the radio button was not selected, then the participant was removed from the data set. The attention check was passed by 233 adults (*M* = 33.8 years, *SD* = 9.5 years, 39% female). These data were used to check the reliability of the ethical attitudes questionnaires. Two people did not disclose gender, and so their data was removed as we were interested in gender effects. Finally, analysis of food ratings were carried out using data from participants who stated they would sample the relevant food if they had a chance to taste it in a supermarket – in order to avoid those participants who do not like the specific foods influencing the data. Six participants would not have eaten any of the three foods, and so were removed from the overall data set. The remaining sample contained 225 adults (*M* = 33.7 years, *SD* = 9.4 years, 37% female). See Table 1 for the summary demographics of the final sample.

**Table 1:** Descriptive statistics for the demographics of the final sample in Study 1.

	Mean	*SD*
Age (years)	33.8	9.4
Gender	39.1% female	
Income to nearest £1000	£36,000	£24,000
Dietary preference	Omni = 179	
	Flexi = 22	
	Poll = 5	
	Pesc = 6	
	Ovo = 10	
	Vegan = 1	
	Other = 2	
Animal attitudes	3.51	0.75
Ethical attitudes	1.44	0.33
Environmental attitudes	3.08	0.48
Combined Attitude	8.03	1.19
Hunger	2.80	1.90
Tiredness	3.48	2.03

#### Design

A within-subjects factorial design was employed, with all participants experiencing all conditions. The factors were ethical valence of the information provided about the food (i.e., morally good versus morally bad), and whether the ethical information might be expected to leave a trace or not on the food (e.g., organic condition versus high wages condition). For each food type a control and two manipulation vignettes were created.

#### Vignettes

Vignettes were created concerning three foods – chocolate, lobster, and orange juice. See Table 2 for a summary of the vignettes, and Supplementary Material for the precise wording. Chocolate was chosen as it often available in organic and Fairtrade versions. For chocolate we created two different positive manipulations and a control condition in order to assess if positive information would increase expectations of the food. One of the positive manipulations (organic) was anticipated to leave a trace on the food, and one of the positive manipulations (high wages) was not anticipated to leave a trace on the food. This allowed us to separately examine the effect of positive information *per se*, and the effect of positive information that would leave a trace, on expectations of that food. We expected the positive manipulations to enhance people’s expected experience of eating chocolate and the trace manipulation to enhance expected experience to a greater extent than the non-trace manipulation.

**Table 2:** Summary of vignettes presented to participants in Study 1.

	Chocolate	Lobster	Orange Juice
Control	Control	Control	Control
Positive trace	Organic	Humanely killed	
Positive no trace	High wages	Sustainably fished	
Negative trace			Non-organic production
Negative no trace			Coal-heated greenhouses

Lobster was chosen in order to explore the effect of giving information about the killing of an animal and because we suspected that, in line with the Liao and Meskin (2018) prediction, positive ethical information with a trace might have a negative effect on expectations in this case. Several studies indicate that crustaceans are able to feel pain (Elwood et al., 2009; Elwood, 2012) leading to controversy about the way in which lobsters are killed before cooking. For lobster we created only positive vignettes (control, positive/no trace – sustainable, positive/trace – humanely killed).

Finally, orange juice was chosen in order to look at the effects of negative information on a product that is perceived as healthy. Again, we examined the effect of trace (control, negative/trace – non-organic, negative/no trace – high greenhouse gas emissions). We expected the negative information to have a negative effect on peoples expected experience of consuming orange juice and the trace manipulation to have a greater negative effect than the non-trace manipulation.

#### The Survey

The survey was hosted on Qualtrics and consisted of two sections. In the first section participants read each vignette, and then rated their expectations of the product described therein. The order of the vignettes was randomized (order determined by Qualtrics software) and every participant read every vignette. Expectations of each product were rated on four attributes, adapted from the Food Choice Questionnaire (FCQ; Steptoe et al., 1995); participants were asked to indicate the extent to which they expected the product to: be delicious, be good, be nutritious, make me feel good. Ratings were made on a visual analog scale, from “Definitely do not expect” to “Definitely expect.” This was converted into a 0 – 100 scale. These product ratings formed the dependent variables.

The second section consisted of a number of questions compiled from existing surveys, measured on Likert scales which measured ethical attitudes and motivations toward animal rights – five items from the Animal Attitude Scale (Herzog et al., 2015; 1 – 7 Likert scale), environmental issues – four items from the Ecoscale (Stone et al., 1995; 1 – 5 Likert scale) and the Ethical self-identity scale (Michaelidou and Hassan, 2008; 1 – 7 Likert scale). Visual analog scales of hunger and fatigue (see Schuldt and Hannahan, 2013) were included as control variables. Basic demographic information including age, gender, dietary restrictions (e.g., vegan) and household income were also recorded. Participants were asked if they would be willing to try each food described in the vignettes if they were available to sample in a supermarket. An attention check question was included within the survey to ensure participants were reading the questions.

#### Procedure

Participants were recruited via an advertisement on Amazons Mechanical Turk. To avoid social desirability compromising the implicit element of the study (see Puska et al., 2018) the purpose of the study was not presented in the advertisement. Once they agreed to take part in the study, participants were directed to a survey which was hosted on Qualtrics. Each participant was asked to read a brief text and provide consent before reading the nine vignettes and rating their expectations of each product. Participants then completed the second section of the survey which addressed ethical attitudes and motivations, demographic information and food preferences. All participants were debriefed. Participation in the study took approximately 7 min. Ethical approval for the study was granted by University of Leeds, School of Psychology ethics committee, date 8/11/2017, reference PS 108. Participants were able to withdraw their data up to 1 week following participation. Their data were identifiable via self-generated pseudonyms.

#### Data Analysis

Data were analyzed using R version 3.5.0 with the following packages: tidyverse (Wickham, 2017) to organize the data, psych (Revelle, 2018) for checking the reliability of the questionnaire, lme4 (Bates et al., 2015) and lmerTest (Kuznetsova et al., 2017) for carrying out multilevel analysis.

Data were analyzed using multilevel analysis, with random factor of participant. This was the optimal way of analyzing these data as it deals well with repeated measures and with missing data. Data was excluded if a participant failed the attention check. Ratings were removed for foods which people would not sample if they were given it for free in a supermarket.

A *z*-score was calculated for income. Cronbach’s alpha was calculated for the ethical attitude scales, to check that the scales could be combined to create one new variable. A *z*-score for this new variable was then calculated. Correlations between the participant ratings of deliciousness, goodness, feel good and nutritiousness were calculated, to determine if the scores were independent. Cronbach’s alpha was calculated to see if the responses could be combined to create a single dependent variable. The *z*-score of the combined responses was used as the DV.

Analysis was conducted separately for each food. An initial model was created with fixed factors of age, gender, hunger, tiredness, income, valence, trace, and ethical attitudes. Factors which did not contribute to the model were removed. Interactions between ethical attitudes and trace, and attitudes and valence, were added in order to address the specific hypotheses that were assessed for each food. Models were compared using likelihood ratio tests. An alpha of 0.05 was adopted throughout.

### Results

Data from 225 participants was examined for answers to the questionnaire. There were no missing data. The questions which were taken from the Animal Attitude Scale (Herzog et al., 2015; 1 – 7 Likert scale), the Ecoscale (Stone et al., 1995; 1 – 5 Likert scale) and the Ethical self-identity scale (Michaelidou and Hassan, 2008; 1 – 7 Likert scale) were rescaled to be out of 1, in order to avoid any issues of the differences in the Likert scales. Negatively scored questions were reversed. The three scales were summed to create an overall attitude score. The Cronbach’s alpha for the 11 ethical items was 0.75 (95% CI 0.70–0.79). This is acceptable, so the combined ethical attitude score was used in all subsequent analyses.

The ratings for the four attributes (delicious, makes me feel good, be good, nutritiousness) were all highly correlated (minimum *r* = 0.53). Generally people are poor at rating attributes independently (Thorndike, 1920). Therefore, the ratings for the attributes were combined by summing the score for each quality. The Cronbach’s alpha for the ratings was 0.87 (95% CI 0.86–0.88). The *z*-score of this new variable was used as the dependent variable in all subsequent analysis.

The means and standard deviations for the ratings can be seen in Table 3. It is worth noting that many participants were excluded in the lobster case, as they would not have sampled lobster in the supermarket. Positively valenced manipulations improved the expected experience of the products, compared with the control condition, and negative manipulations decreased the expected experience of the products. In the negatively valenced case, there is little obvious difference between the trace condition and the non-trace condition. In one positive case, chocolate, the trace condition increases expectations of the product compared to the non-trace condition. Conversely, for lobster, the trace condition led to lower expectations than the non-trace condition.

**Table 3:** Means and standard deviations of the *z*-scores of the participant ratings derived from the ratings of expected deliciousness, nutritioness, goodness and feel good, for the vignettes in Study 1.

Food	*N*	Condition	Mean	*SD*
Chocolate	219	High worker wages	0.12	0.86
		Organic	0.72	0.75
		Control	−0.26	0.77
Lobster	113	Sustainably fished	0.71	0.77
		Humanely killed	0.53	0.82
		Control	0.26	0.78
Orange juice	210	Coal-heated greenhouses	−0.55	1.05
		Non-organic production	−0.64	0.95
		Control	−0.22	0.97

#### Multilevel Modeling

##### Chocolate

In the initial model, hunger, tiredness, income, age and sex failed to contribute to the model. There were significant effects of trace [*F*(1,438) = 88.90, *p* < 0.0001], valence [*F*(1, 438) = 37.78, *p* < 0.0001], and attitudes [*F*(1,219) = 4.66, *p* = 0.032]. A second model was constructed with only the significant predictors, and a third model was constructed with the interactions between attitudes and trace, and the interactions between attitudes and valence. This third model was significantly better than the second model with no interactions [χ^2^(2) = 30.78, *p* < 0.0001]. In the final model, the trace by attitudes interaction was significant [*F*(1,438) = 22.98, *p* < 0.0001], as were the effects of valence and trace. The fixed coefficients are shown in Table 4. Both positive valence and trace increased the rating of the expected experience of chocolate. Those who scored higher in ethical attitudes generally had lower expectations of the chocolate. However, people with higher ethical attitudes had higher expectations of the chocolate with a positive trace than those people who scored lower on the ethical attitudes measures.

**Table 4:** Estimated fixed effects (and standard errors) from linear mixed model fitted by maximum likelihood, for the three foods used in Study 1.

	Chocolate (*N* = 219)	Lobster (*N* = 113)	Orange juice (*N* = 210)
Intercept	−0.262 (0.052) ^∗∗∗^	0.246 (0.072) ^∗∗∗^	−0.101 (0.080)
Positive valence	0.387 (0.061) ^∗∗∗^	0.452 (0.070) ^∗∗∗^	–
Negative valence	–	–	−0.376 (0.046) ^∗∗∗^
Trace	0.592 (0.061) ^∗∗∗^	−0.187 (0.070) ^∗∗^	–
Attitudes	−0.199 (0.052) ^∗∗∗^	−0.198 (0.068) ^∗∗^	−0.233 (0.118) ^∗∗∗^
Attitudes × Positive Valence	0.011 (0.060)	–	–
Attitudes × Trace	0.288 (0.060) ^∗∗∗^	–	–
Sex (Female)			−0.279 (0.118) ^∗^

*^∗^p < 0.05; ^∗∗^p < 0.01; ^∗∗∗^p < 0.001. Tests of significance by t-tests using Satterthwaite’s method.*

##### Lobster

In the initial model, hunger, tiredness, income, age, and sex failed to contribute to the model. There were significant effects of trace [*F*(1,226) = 7.18, *p* = 0.008], valence [*F*(1,226) = 42.00, *p* < 0.0001], and attitudes [*F*(1,113) = 7.68, *p* = 0.007]. A second model was constructed with only the significant predictors, and a third model was constructed with the interactions between ethical attitudes and trace, and the interactions between attitudes and valence. This third model was not significantly better than the model with no interactions [χ^2^(2) = 2.50, *p* = 0.29], suggesting that ethical attitudes had little interaction with the manipulations of value for lobster. The fixed coefficients are shown in Table 4. Both positively valenced manipulations were rated higher overall. This appears to conflict with our prediction that in this case ethical manipulations which involve a trace would lower expectations. However, the manipulation which involved the presence of a trace on the product, ‘humane killing,’ resulted in a lower rating than did the non-trace manipulation, ‘sustainable fishing.’ This might simply be because people value sustainability more than the humane treatment of animals in this case. But it also might reflect some negative effect of trace.

One might worry whether there really is no trace in the ‘sustainable fishing’ case. After all, it might be thought that the use of traps which let young lobsters escape would leave a trace since the remaining lobsters would tend to be (detectably) older and bigger than in the control condition. The concern here reinforces the need to do more theoretical and empirical work on the notion of trace. But we are unconvinced that there is a clear case of trace in this condition. It does not seem to be the case that that the specific lobsters participants imagine eating in the sustainable condition would have likely been younger and/or smaller if production methods had been different. (Although they might have eaten different lobsters). At a minimum, this is very unlike a paradigmatic case of trace such as the cruel treatment of geese in traditional gavage-based foie gras. In that case, the specific item of food bears the trace of its production since that very bit of food would have been perceptibly different had it been produced differently. But, again, we think there are significant and complicated issues worth investigating here.

##### Orange juice

In the initial model, hunger, tiredness, income, age and trace all failed to contribute to the model. There were significant effects of valence [*F*(1,420) = 38.30, *p* < 0.0001], attitudes [*F*(1,210) = 17.2, *p* < 0.0001], and sex [*F*(1,210) = 6.15, *p* = 0.014]. A second model was constructed with only the significant predictors, and a third model was constructed with the interactions between attitudes and trace, and the interactions between attitudes and valence. This third model was not significantly better than the model with no interactions [χ^2^(2) = 2.27, *p* = 0.32], suggesting that ethical attitudes had little interaction with the manipulations of value for orange juice. The fixed coefficients are shown in Table 4. The negative valence reduced the rating of the expected experience for orange juice, and those with higher ethical attitude scores rated orange juice lower overall. Females also rated orange juice lower overall.

### Discussion

This study clearly demonstrates that ethical information has an effect on the expectations of products. For all three foods, there was an effect of ethical information. Positive information increased ratings for lobster and chocolate, while negative information decreased ratings of orange juice. For positive information, there was an effect of trace, but the effect of trace depended upon the specific manipulation. For chocolate, the trace condition was ‘organic’ which increased ratings of the food. Conversely, the trace condition for lobster which was ‘humane killing,’ resulted in a lower rating than did the non-trace ethical condition, ‘sustainable fishing.’ In both cases, ethical manipulations improved expectations. But they did so less in the trace condition. There are a number of hypotheses that are consistent with this result. One possibility is that people value sustainability more than the humane treatment of animals in some cases. Another possibility is that trace itself is a relevant factor. Note that these two hypotheses are consistent with one another—people may value sustainable fishing more than humane treatment in this case because they believe that the trace produced by the latter detracts from the overall effect of the moral condition. Ethical values had an influence on ratings. Across all three products, those who had higher ethical values generally rated the foods lower. For chocolate, higher ethical values were associated with a bigger effect of both trace and positive valence.

In order to investigate whether effects that were found in an online survey reflected the actual experience of eating a product, we carried out Study 2.

## Study 2

The second study aimed to extend the findings of Study 1 and explore how ethical information influences consumer product experience. In order to fully explore how ethical information of differing valence and content influences consumer experience it was necessary to limit the number of products used. Chocolate was chosen as Study 1 demonstrated that consumer expectations of chocolate were influenced by positive ethical information, and that ethical values were important for this product. We aimed to extend the findings of Study 1 and explore how both positive and negative ethical information influence consumer chocolate perception.

Following on from Study 1 we hypothesized that ethical information would influence the experience of eating chocolate, and this would be reflected in the ratings of the chocolate. If experience is similarly affected by ethical information as the expectation of eating, then the different ethical manipulations (e.g., organic, worker pay) should affect whether the experience of eating the chocolate differs. Specifically, we expected that valence of the ethical manipulation (e.g., positive, negative) would be important, as would whether the manipulation would be expected to leave a trace. We also expected that these effects might depend upon the ethical attitudes of consumers.

### Methods

#### Participants

Sixty-three students and staff (27.8 years, *SD* = 8.8, 68% female) were recruited from University of Leeds, United Kingdom. A minimum sample size *N* = 34 was determined for power of 80%, with *d* = 0.5 (a medium effect size) and alpha = 0.05, using an *a priori* sample size calculator (Faul et al., 2009). All subjects were given a course credit or a chocolate bar for their participation. An attention check was included in the questionnaire, which was identical to that described for Study 1. Participants who did not pass the manipulation check were excluded from subsequent analysis (*N* = 9). Across the food ratings, 17 participants did not answer every item (total missing data = 5.7%). Participants who missed more than 25% of the items were excluded (*N* = 3). One participant did not answer every attitude item, and their data were excluded. The demographics of the final sample (*N* = 50) are shown in Table 5.

**Table 5:** Demographics of the final sample in Study 2.

	Mean	*SD*
Age	27.04	8.33
Gender	38 (76%) female	
Income to nearest £1000	£31,000	£31,000
Dietary preference	Omni = 28	
	Flexi = 12	
	Poll = 1	
	Pesc = 1	
	Ovo = 5	
	Vegan = 2	
	Other = 1	
Animal attitudes	3.67	0.42
Ethical attitudes	1.51	0.27
Environment attitudes	2.30	0.35
Combined attitude	7.48	0.55
Hunger	1.65	1.75
Tiredness	3.06	1.95

#### Ethics Statement

The study was approved by the School of Psychology, University of Leeds, ethics committee on 13/02/2018, reference PS 283.

#### Design and Materials

A within-subjects factorial design was employed. The factors were vignette information valence (positive vs. negative) and type (organic vs. worker conditions). A control and four manipulation vignettes which were presented (see Supplementary Materials). These were: positive which would leave a trace (organic), negative which would leave a trace (non-organic), positive which would not leave a trace (high wages) and negative which would not leave a trace (low wages).

A commercially available dark chocolate was purchased for the study (Co-op, United Kingdom). All branded packaging and foil was removed, and the chocolate bar was not marked with any branding or logos. Each chocolate bar was broken into square pieces. The pieces of chocolate were then placed in five small containers and labeled A, B, C, D, and E. Five labeled samples of chocolate were prepared for each participant. Three neutral flavored crackers (water biscuits) were broken into pieces and placed in a small bowl. In addition, each participant was provided with a glass and small jug of water.

A survey similar to that described in Study 1 was employed. The survey was presented in two sections. The first part presented the vignettes and attribute rating scales. Presentation order of the vignettes was randomized. Each sample was evaluated on four attributes (is delicious, is good, is nutritious, makes me feel good), and the rating of each attribute was indicated on a visual analog scale (Strongly agree-Strongly disagree). The second section of the questionnaire consisted of the ethical attitudes survey, control scales and demographic questions (see Study 1). The survey was hosted on Qualtrics and completed on a laboratory desktop computer.

#### Procedure

Before attending the laboratory session, participants completed a screening questionnaire. Participants with a dislike of chocolate, an allergy, or intolerance to a specific ingredient in the chocolate or crackers, were excluded from the study. The experimenter contacted ineligible participants and explained that they would not be able to take part in the laboratory study.

Participants who were able to take part in the study were allocated a laboratory session timeslot. The study was conducted on an individual basis in a laboratory cubicle. To avoid compromising the implicit element of the study, the full purpose of the study was not declared. Upon entering the cubicle participants were seated at a computer, next to a white plastic tray which contained the five labeled (A–E) containers of chocolate, a small bowl of crackers, the water jug and glass.

The experimenter then directed the participants to the survey. Participants were informed that they would be presented with information (a vignette) about each chocolate sample. Participants were instructed to read the information about the chocolate sample, before tasting the product. Having tasted the sample, they were instructed to rate the sample on the four attributes. Participants were asked to eat a small piece of cracker and drink some water between each chocolate sample as a palette cleanser. This process was repeated for each of the five chocolate samples. The order was randomly determined by Qualtrics.

Participants then completed the second section of the survey, which consisted of the ethical attitudes survey, control scales and demographic questions (see Study 1). In order to find out if participants had guessed the purpose of the study, the final item on the questionnaire asked participants to describe the hypotheses beyond those described in the study brief. Only 4 participants guessed the purpose, so suspicion about the study was not a concern. Once the participant indicated that the survey was completed they were debriefed. Participation in the study took approximately 25 min.

#### Data Analysis

The same data analysis approach was used as in Study 1. Two sets of multilevel models were created. The first set compared the effect of the manipulations to the reference control category. Interactions with ethical values were examined in a second model, to see if ethical values affected the effect of the ethical manipulations on the experience of eating chocolate. The second set of models excluded the control case, and examined whether there was any effect of manipulations that would leave a trace, and if that interacted with the valence of the manipulations. Models were compared using likelihood ratio tests, and alpha of 0.05 was adopted throughout.

### Results

Correlations between the ratings for the attributes ranged from *r* = 0.33 to *r* = 0.76. Cronbach’s alpha for the rated attributes was 0.83 (95% CI 0.79 – 0.86), so they were combined into a single score, as in Study 1. Where the rating for an attribute was missing, the mean was calculated across the remaining ratings of that participant. The z-score of the mean rating was used as the dependent variable. Cronbach’s alpha for the ethical scales was low at 0.39 (95% CI 0.15 – 0.64). Omitting items did not meaningfully improve the scale, suggesting that in this smaller sample the ethical attitudes scale was not unidimensional. We therefore decided to omit the ethical attitudes scale from further analysis in the laboratory study.

Both the high wage (*M* = 0.286, *SD* = 0.939) and the organic (*M* = 0.392, *SD* = 0.953) conditions were rated higher than the control (*M* = −0.009, *SD* = 1.027) condition. Similarly, the low wage (*M* = −0.453, *SD* = 0.873) and the non-organic (*M* = −0.216, *SD* = 0.988) conditions were rated lower than the control condition. Therefore, positive manipulations improved the ratings, while negative manipulations reduced the ratings. Ratings were lower in the low wage (would not leave a trace) condition than in the non-organic condition, but higher in the organic (would leave a trace) than high wage conditions, suggesting that trace had an inconsistent effect.

The initial model examined the effect of the manipulation, hunger, tiredness, income, age and sex. The only significant factor was manipulation [*F*(4,188) = 19.51, *p* < 0.0001]. In order to investigate the effect of manipulation further, a new model was created in which each manipulation was coded as to whether there was a trace or not (organic, non-organic coded for trace), and for valence (positive, negative), and the interaction between valence and trace was included. Responses to the neutral control condition were excluded. This demonstrated effects of both valence [*F*(1,150) = 73.48, *p* < 0.0001] and trace [*F*(1,150) = 4.78, *p* = 0.03], but no interaction between them (*F* > 1). The fixed coefficients are shown in Table 6.

**Table 6:** Estimated fixed effects (and standard errors) from linear mixed model fitted by maximum likelihood, for the two models in Study 2.

	Manipulation	Trace and valence
Intercept	0.008 (0.134)	−0.453 (0.131) ^∗∗∗^
High wage	0.294 (0.106) ^∗∗^	
Low wage	−0.445 (0.106) ^∗∗∗^	
Non-organic	−0.207 (0.106)	
Organic	0.400 (0.106) ^∗∗∗^	
Trace		0.238 (0.111) ^∗^
Positive valence		0.739 (0.111) ^∗∗∗^
Trace × Positive valence		−0.132 (0.157)

*The first model included the factor of manipulation, with levels including all five ethical vignettes. The second model classified the manipulations according to whether they would be expected to leave a trace on the food, and the valence of the manipulation. ^∗^p < 0.05; ^∗∗^p < 0.01; ^∗∗∗^p < 0.001. Tests of significance by t-tests using Satterthwaite’s method.*

### Discussion

The results of the second study suggest that ethical information does affect the experience of eating chocolate. Positive information increased the ratings of the experience of eating chocolate. There was no interaction with trace, but there was a main effect of trace. This needs careful interpretation, as it suggests that, overall, having a trace improves the experience of eating chocolate. That is, chocolate with a positive trace was rated higher than chocolate without a trace, but similarly, chocolate with a negative trace was also rated higher than chocolate without a trace. The results of the multilevel modeling reflect the mean ratings that are reported. Non-organic (a negative manipulation that would not leave a trace) is not rated differently to control.

## General Discussion

We found that the presence of ethical information affected both consumer expectation and experience of food. The effect of ethical information on expectations of foods was affected by valence, whether the manipulation might be expected to leave a trace and food type. The valence of ethical information affected consumer expectation and experience of food, with positive information increasing, and negative information decreasing, ratings given by participants.

The effect of trace was mixed. For chocolate expectation, a manipulation that would be expected to leave a trace, but which is positive, produced a bigger improvement in expected eating experience than one which would not leave a trace. However, this was not found when we examined actual consumption. This is in line with previous research by Bratanova et al. (2015) which similarly finds that a food’s ethical origins do not always translate into enhanced experience. For lobster, both sustainably caught and humanely killed lobsters were rated more favorably than the control condition. The finding that the humanely killed lobster is rated less favorably than the sustainably caught one could be cautiously interpreted as support for the contextualist view put forth by Liao and Meskin (2018). Following their line of thought about the ikizukuri sashimi case, a direct prediction of Liao and Meskin (2018) might be that the humanely killed lobster would be rated less favorably than in the control condition. We did not find this, but it may be that the general positive framing of the vignette may have ameliorated that effect. We have discussed other complications with this case above. That the humane killing case is judged less favorably than a similarly presented positive case is worthy of further investigation.

Overall, we observed that trace only affected expectations of products when manipulations were presented with positive ethical information. The effect of trace was absent when the ethical information was negative.

In contrast with other studies (Lee et al., 2013; Bratanova et al., 2015; Sörqvist et al., 2015), we found only a limited role for ethical values. Those with higher ethical values gave lower ratings for the expected experience of eating chocolate. In addition, we observed an interaction between ethical attitudes and trace. Participants with higher ethical values gave higher ratings to chocolate where the manipulation might leave a trace than to the manipulation which would not leave a trace. However, the ethical scale we used was not reliable in the second study, so this needs cautious interpretation.

In line with existing research (Schuldt and Schwarz, 2010; Bratanova et al., 2015) we found that information regarding a food’s ethical status influenced consumer experience. Echoing the findings of Enax et al. (2015), we found that both expectations and experience of chocolate can be improved by describing the product as ethical.

We assessed how negative ethical information impacts product experience. Though we only explored the impact of negative information in a limited number of scenarios, negative ethical information influenced product experience (chocolate in Study 2) and expectation (orange juice in Study 1). This provides a valuable contribution to the small number of studies which have explored negative information (e.g., Bratanova et al., 2015; Anderson and Barrett, 2016). Further to previous research which primarily focused on the impact of negative ethical information on sensory attributes (e.g., taste), the current research indicates that the impact of negative ethical information is not limited to specific features or characteristics of foods.

### Limitations

A low Cronbach’s alpha was observed for the ethical attitude scales in Study 2. The relatively small sample size may have contributed to this. Therefore, it is unclear what role ethical attitudes play in product experience. Study 1 lacked balance, with only one product (orange juice) being presented with negative ethical information. Presenting negative information with other foods would provide additional understanding of impact which valence can have upon product expectations.

Recruitment for Study 1 was conducted via mTurk and limited to participants based in the United Kingdom which attracts a limited demographic. Similarly, participants for Study 2 consisted of staff and students from a university in the United Kingdom. Though the sample is sufficient for proof-of-principle, recruiting participants from a wider demographic would be beneficial (Bardi and Zentner, 2017). In addition, as cultural differences can influence attitudes toward ethically produced foods (Sörqvist et al., 2016) and taste preferences (Poelman et al., 2008), it is possible that replicating the current study in a different country or region may yield different results.

Only a limited number of foods (e.g., chocolate, orange juice) and types of ethical information (e.g., organic, worker wages) were used within the study. Further replication is required which will examine a wider variety of foods and manipulations.

### Implications

The current research offers some intriguing findings and insight into methods which could contribute to a shift in food choice and consumption. Such methods may might even play a role in potential solutions to growing concerns about food security and environmental sustainability.

We have demonstrated that positive ethical information can increase consumer expectations of a product. Since expectations of nutrition, taste and quality can influence consumer purchase decisions (Furst et al., 1996) companies might usefully communicate ethical information (e.g., that products are sustainably produced) when marketing their products. In turn, this may increase consumer expectations of their products, resulting in increased sales of ethical foods. As consumer choice has been highlighted as a contributing factor to food systems and environmental sustainability (Clark and Tilman, 2017), we propose that the halo effect of ethical information could be utilized to drive change within the market, toward more environmentally sustainable food practices. However, this approach does not come without risk. As observed in Study 1, certain forms of positive ethical information are more effective than others. The current research found that a positive trace manipulation may not always be the most effective at increasing the expectation of some foods, as in some cases a moral flaw might increase the deliciousness of a food, as suggested by Liao and Meskin (2018).

### Future Research

Based on existing literature and our findings, we suggest three areas for future research. First, we used a single product, chocolate, to explore the impact of ethical information on food perception (Study 2). The expected effect of positive-trace information on product ratings was observed, yet, the data did not reveal an effect of ethical attitudes on product experience. Hence, it is unclear whether the role of ethical attitudes is limited to expectation of foods, or whether this effect is an artifact of the chosen food, chocolate. Repeating the methods of Study 2 with a range of foods (e.g., orange juice, vegetables, and potato chips) would answer this question.

Second, the current research investigated how ethical information influences expectation and perception of a product on four attributes (delicious, feel good, is good, nutrition). However, all attributes were similarly influenced by the ethical information. This contrasts with previous literature which reported that different attributes can be independently influenced (Schuldt and Hannahan, 2013). By exploring the impact of ethical information on additional sensory attributes (e.g., texture, flavor strength, color), the parameters of trace and valence could be further explored.

Finally, in order to test the proposal that moral flaws make some foods delicious (Liao and Meskin, 2018) and gain future understanding of the impact of trace, future research could assess how consumer expectations and perception of foods such as ikizukuri sashimi or crate-raised veal are influenced by ethical information varying by valence and trace. This may reveal cases in which negatively valenced trace information leads to an increase in product expectations and experience.

### Conclusion

The results of our two studies support the finding that ethical information can affect expectation across a range of foods, and that the valence of this information is a significant factor in both expectation and experience. We also replicated earlier findings which suggested that ethical attitudes have a greater effect on expectations of food than on the experience of food. Inspired by recent work in philosophical aesthetics, which suggests that information about ethically relevant factors in production that leave a trace on a food item may affect consumers differently than information about factors which do not leave such a trace, we explored the significance of this feature. Our results suggest that the trace factor is significant: ethically relevant features which might be expected to affect the perceptual experience of a food item had a different effect on expectation and experience than did ethically relevant features which might not be expected to leave a trace We also found that in one case, the effect of a morally positive manipulation which might be expected to leave a trace on the food had less of a positive effect than did a positive manipulation that might not be expected to leave a trace. Our results, then, provides limited support for the contention of some philosophers that the effect of ethical factors can vary depending on contextual factors such as *type of ethical factor* and *type of food*.

## Ethics Statement

This study was carried out in accordance with the recommendations of School of Psychology ethics committee, University of Leeds with written informed consent from all subjects. All subjects gave written informed consent in accordance with the Declaration of Helsinki. The protocol was approved by the School of Psychology ethics committee, University of Leeds. Reference numbers: 13/02/2018, reference PS 283; 8/11/2017, reference PS 108.

## Author Contributions

All authors participated in design of the studies. BA created the studies and led the data collection. PB-B carried out the data analysis. All authors participated in manuscript preparation and revised the final version of the manuscript.

## Conflict of Interest Statement

The authors declare that the research was conducted in the absence of any commercial or financial relationships that could be construed as a potential conflict of interest.

## Footnotes

1 Payment was increased in increments ($.20, $.50, $.80 to $1.00) due to low initial uptake.
