# Supplementary material for: Delicious but Immoral? Ethical Information Influences Consumer Expectations and Experience of Food
Source: Front Psychol. 2019 Apr 24;10:843. doi: 10.3389/fpsyg.2019.00843 (PMC6499174; doi:10.3389/fpsyg.2019.00843)
Supplement: Supplementary file 1 [file Data_Sheet_1.PDF]

Supplementary material: the vignettes which were used in Study 1 and Study 2.

Study 1

Chocolate

Control

This brand of chocolate provides farmers with an average market price for their produce. The workers receive a typical wage and the characteristic basic rights for the region. The brand source products from producers which rely on standard agricultural chemicals. This includes the use of common pesticides, herbicides and oil-based fertilisers which can contribute to agriculture's greenhouse gas emissions.

Organic (positive, trace)

This brand of chocolate provides farmers with an average market price for their produce. The workers receive a typical wage and the characteristic basic rights for the region. The brand source products from producers which rely on organic farming methods. The products are produced using natural ways to control pests and disease rather than synthetic pesticides and herbicides. The producers use organic rather than oil-based fertilisers which can significantly reduce agriculture's greenhouse gas emissions.

High Wages (positive, no trace)

The brand of chocolate provides an above average market price for the farmers' produce. The workers receive a better wage and more basic rights than is typical in the region. This brand of chocolate aims to increase standards of living for farmers and workers. The brand source products from producers which rely on standard agricultural chemicals. This includes the use of common pesticides, herbicides and oil-based fertilisers which can contribute to agriculture's greenhouse gas emissions.

Lobster

Control

This restaurant sells lobsters which are caught using typical methods. The company which sources lobsters for the restaurant use traps which are common in the lobster industry. The traps catch lobsters of all ages and sizes, which may decrease the sustainability of the industry. The lobsters are killed and cooked by being put into a pan of boiling water. This method is common within the restaurant industry.

Sustainably fished (positive, no trace)

This restaurant sells sustainably caught lobsters. The company which sources the lobsters for the restaurant use traps which let younger lobsters escape, with only older lobsters remaining in the traps. This allows the younger lobsters to grow and reproduce several times before being caught, contributing to the sustainability of the industry. The lobsters are killed and cooked by being put into a pan of boiling water. This method is common within the restaurant industry.

Humanely killed (positive, trace)
This restaurant sells lobsters which are caught using typical methods. The company which sources lobsters for the restaurant use traps which are common in the lobster industry. The traps catch lobsters of all ages and sizes, which may decrease the sustainability of the industry. The lobsters are killed using the quickest and most humane method. A knife is used to kill the lobster before it is cooked. This method aims to minimise any unnecessary distress to the animal.

Orange Juice
Control
This brand of orange juice sources oranges from a producer which relies on standard agricultural chemicals. This includes the use of common pesticides, herbicides and oil-based fertilisers which are normally used in agriculture. The oranges are grown in greenhouses which are heated using a combination of fossil fuels and solar power. This method produces typical levels of carbon dioxide for agriculture, this contributes to greenhouse gases and global warming.

Non-organic production (negative, trace)
This brand of orange juice sources oranges from a producer which relies heavily on agricultural chemicals. This includes the intense use of pesticides, herbicides and oil-based fertilisers which can affect the environment by poisoning and contaminating water courses, and disrupting ecosystems. The oranges are grown in greenhouses which are heated using a combination of fossil fuels and solar power. This method produces typical levels of carbon dioxide, this contributes to greenhouse gases and global warming.

Coal-heated greenhouses (negative, no trace)
This brand of orange juice sources oranges from a producer which relies on standard agricultural chemicals. This includes the use of common pesticides, herbicides and oil-based fertilisers which are commonly used in agriculture. This brand of orange juice is not concerned about producing climate-friendly food. The producers are heavily dependent on the use of fossil fuels. The oranges are grown in coal-heated greenhouses, emitting high levels of carbon dioxide, this greatly contributes to greenhouse gases and global warming.

Study 2
Control
This brand of chocolate provides farmers with an average market price for their produce. The workers receive a typical wage and the characteristic basic rights for the region. The brand source products from producers which rely on standard agricultural chemicals. This includes the use of common pesticides, herbicides and oil-based fertilisers which can contribute to agriculture's greenhouse gas emissions.

Organic (positive, trace)
This brand of chocolate provides farmers with an average market price for their produce. The workers receive a typical wage and the characteristic basic rights for the region. The brand

source products from producers which rely on organic farming methods. The products are produced using natural ways to control pests and disease rather than synthetic pesticides and herbicides. The producers use organic rather than oil-based fertilisers which can significantly reduce agriculture's greenhouse gas emissions.

High wages (positive, no trace)
The brand of chocolate provides an above average market price for the farmers’ produce. The workers receive a better wage and more basic rights than is typical in the region. This brand of chocolate aims to increase standards of living for farmers and workers. The brand source products from producers which rely on standard agricultural chemicals. This includes the use of common pesticides, herbicides and oil-based fertilisers which can contribute to agriculture’s greenhouse gas emissions.

Low wages (negative, no trace)
The brand of chocolate provides a below average market price for the farmers’ produce. The workers receive a lower wage and fewer rights than is typical in the region. This brand of chocolate is not concerned with increasing the standards of living for farmers and workers. The brand source products from producers which rely on standard agricultural chemicals. This includes the use of common pesticides, herbicides and oil-based fertilisers which can contribute to agriculture’s greenhouse gas emissions.

Non-organic (negative, trace)
This brand of chocolate provides farmers with an average market price for their produce. The workers receive a typical wage and the characteristic basic rights for the region. The brand source products from producers which rely heavily on agricultural chemicals. This includes the use of pesticides, herbicides and oil-based fertilisers which can contribute to agriculture’s greenhouse gas emissions and affect the environment by poisoning and contaminating water courses, and disrupting ecosystems.
